# Design of a Felid-like Humanoid Foot for Stability Enhancement

**DOI:** 10.3390/biomimetics7040235

**Published:** 2022-12-12

**Authors:** Zhaoyang Cai, Xuechao Chen, Qingqing Li, Huaxin Liu, Zhangguo Yu

**Affiliations:** School of Mechanical Engineering, Beijing Institute of Technology, Beijing 100081, China

**Keywords:** humanoid robot, bionic, mechanism design

## Abstract

The foot is an important part of humanoid robot locomotion that can help with shock absorption while making contact with the ground. The mechanism of the foot directly affects walking stability. A novel foot mechanism inspired by the toes of felids is proposed. The foot has four bionic modules with soft pads and sharp claws installed at the four corners of a flat foot. This foot can reduce the impact experienced during foot landing and increase the time that the foot is in contact with the ground, which can improve the adaptability of the robot to different ground surface conditions with different levels of stiffness. The main structure of the bionic module is a four-bar linkage consisting of a slide way and a spring. Furthermore, the length of the four-bar linkage and the posture of the claw during insertion into soft ground are optimized to improve the stability and buffering performance. The validity of the proposed foot mechanism has been proved in simulations.

## 1. Introduction

**Figure 1:**
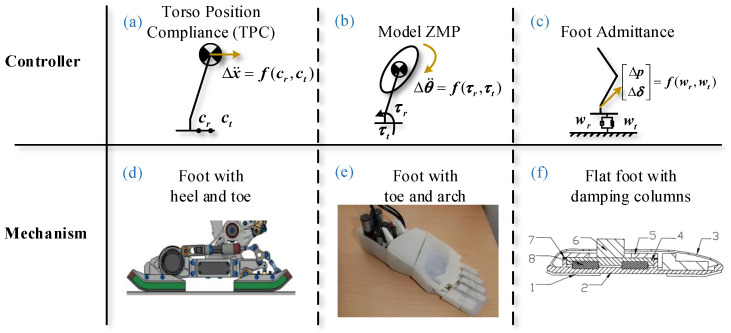
Different strategies for improving stability.

Bionic technology, which integrates the advantages of various natural creatures into human-used machines or tools, has made significant developments [1,2,3,4,5,6,7]. In particular, humanoid robots can replace humans for the completion of some dangerous tasks. A high center of mass (CoM) and small support polygon result in a small stability margin. Therefore, many researchers have focused on determining how to improve the stability of humanoid robot locomotion. As shown in Figure 1, some researchers utilized controllers to improve the stability [8,9,10]. In Ref. [8] as shown in Figure 1a, torso position compliance (TPC) is proposed to modify the center of mass (CoM) using zero-moment point (ZMP) error. In Ref. [9] as shown in Figure 1b, the model ZMP controller uses torso angular acceleration to compensate for ankle torque errors. In Ref. [10] as shown in Figure 1c, admittance controls the foot position and posture so that the real wrench is close to the target wrench. These controllers are based on feedback and usually have the disadvantages of lagging response and limited compensation. Meanwhile, some researchers choose to design functional mechanisms to enhance robot stability [11,12,13]. In Ref. [11] as shown in Figure 1d, a foot with a heel and toe is proposed for anthropomorphic landing and take off. In Ref. [12] as shown in Figure 1e, a foot with a toe and arch is designed to buffer the impact and imitate the launching of the human forefoot. In Ref. [13] as shown in Figure 1f, a flat foot with damping columns is proposed that utilizes rubber to absorb the impact from walking and, thus, improve walking stability. In addition, it does not require additional trajectory planning of the foot and is highly versatile. Prominent examples of legged robots are Asimo from Honda [4], H7 from Tokyo University, HRP-2 from AIST and Kawada Industries, the first running biped QRIO from Sony, Wabian-2R from Waseda University [5], LOLA from Ludwig Maximilians University [11,14], and the Korean robot HUBO from KAIST [3,15,16].

The foot of LOLA has a passive heel with a shock absorber, which contributes to shock attenuation, and a hallux. This special foot can ensure proper ground contact on different surfaces. However, for the hydraulic damper, the relationship between the force and the amount of stretching is not linear. The WABIAN-2R robot achieves a more human-like gait than other humanoids because it can move its waist to extend its knees while walking to avoid singularity. Moreover, the foot has a passive joint for a toe-bending motion. The passive joint is selected as the toe joint based on human gait analysis reports. In this case, the toe muscles are relaxed, and the power for toe motion is seldom supplied to these muscles. However, the robot has no shock attenuation capacity. An anthropomorphic dexterous humanoid foot designed by the Italian Institute of Technology is able to duplicate the three main tasks of the human foot, namely adapting to the contours of the ground, absorbing shock impacts, and storing and releasing energy. BHR-2 is designed with a flexible foot for walking. Rubber bushes and rubber pads are adopted to absorb impact during walking [17]. New foot pads are used in BHR-5 to improve the stability and reliability of the robot. These efforts focused on designing new soles to reduce the walking impact, absorb landing oscillations, and prevent sliding [18]. Pneumat-BB is equipped with a novel robot foot that has a human-like deformable arch and can generate human-like truss and windlass mechanisms [19]. It uses pneumatic actuators and imitates the links, joints, and muscles of the human foot. However, the weight of the pneumatic robot is only 11 kg; therefore, the load performance of the foot needs to be further validated compared with the general humanoid robot. The foot in Ref. [12] is appropriately simplified according to the skeletal structure of the human foot, and the middle part of the foot forms an arch shape that allows the bionic foot to have a certain buffering ability and the ability to store deformation energy. The robotic foot also uses a unique material that provides good cushioning; however, it is less impact-reducing than the human foot.

Early studies on humanoid robot motion planning and control focused on biped locomotion. Although robots have achieved reliable walking abilities, dynamic locomotion is still a challenge compared with that of human beings. There are still many problems in this field, such as fast walking and running, sudden turning, walking on rough terrain, and trajectory generation in complex environments. During the single-leg support period, one leg contacts the ground while the other swings from back to front. For this reason, the humanoid robot rotates around the supporting leg (yaw). The friction between the supporting leg and the ground is the only force to balance the rotation of the swinging leg, which decreases the ability of the robot to walk in a straight line. Considering the impact force from the ground, it is easy for the robot to slide relative to the ground. In addition, when the swing leg contacts the ground, the rigid structure will be damaged by frequent impact, and the mechanism and planning are unable to sufficiently reduce the impact force from the ground [20]. Although the robotic foot aims to replicate the anatomical structure and main functionality of the human foot, the structure of the foot is extremely complex, which increases the probability of error.

Predecessors have spent much time designing robotic feet. It is obvious that the foot is an important part of the humanoid robot. To achieve steady, fast movement of the humanoid robot, it is necessary to design a robot foot with improved stability. One of the keys to stable walking is better grip and buffer capacity. In nature, many animals have the ability to grip and buffer. The felid is a typical example. The foot of a felid has pads and claws, which can reduce impact and improve grip. In Ref. [21], we designed a four-link mechanism fixed on the foot to enhance stability. However, it is not suitable for hard ground because the claw would be damaged by large impacts. To overcome this problem, a multilink underactuated mechanism is proposed, which can reduce the impact from the ground and improve the grip of a robot compared with the original version [21]. The mechanism has springs and links with rational layouts. Improvements to the buffering and stability characteristics can be achieved by designing a reasonable layout of the springs and links and a reasonable mass distribution. Moreover, this optimized design can allow robots to adapt to complex surroundings and continue to move efficiently.

This paper is organized as follows: Section 2 describes the outline of the felid-like foot. Section 3 optimizes the length of the four-bar linkage in the bionic mechanism to ensure the posture of the claw during ground insertion. Section 4 designs a limited structure to improve the ability of the foot to adapt to the ground conditions of differing stiffness levels. Section 5 describes the verification of effectiveness of this foot through simulation. Finally, Section 6 concludes this paper.

## 2. Bionics Design

Reviewing the developments in science and technology, it is easy to find that many major breakthroughs are derived from bionic thought [22]. After millions of years of natural selection and survival of the fittest, the animals that survive are the best. They have a variety of peculiar features that allow them to easily survive in nature. With the development of bionics technology, we found that peculiar biological features are exhibited through the interactions of many factors rather than one single factor.

In nature, cats are hunting experts, some of which stand on the top of the food chain. Cats have evolved to have excellent predation abilities. During hunting, they silently approach their prey and then quickly accelerate. The foot structure of the cat aids it in superbly completing the hunting task. The hunting process relies on the following steps. When the cats approach the target, the sound of the foot contacting the ground is as soft as possible, and the impact is as small as possible. When the cat reaches a point sufficiently close to the target, it needs to achieve maximum speed in minimum time to complete the final kill, and this quick start process requires the foot to have an enormous grip.

The outstanding predation ability of cats is achieved through the interaction of many elements. In addition to robust leg muscles, high-toughness ligaments and delicate bone structures of high toughness, soft “pads” under the foot and elastic claw are also indispensable elements. The “pads” on a cat’s feet have a strong buffering capacity that provides cushioning performance, while the leather of the “pads” protects the foot. The “pads” are able to absorb the impact from the ground. At the same time, the claw retracts to avoid excessive sound and vibration between the ground and the claw. When starting the sprint, the claw roots into the ground and provides adequate grip. Simultaneously, a huge impact from the ground to the soles of the feet is dissipated by plantar “pads”. The “pads” play an important role in protecting the cat’s bones and other parts.

**Figure 2:**
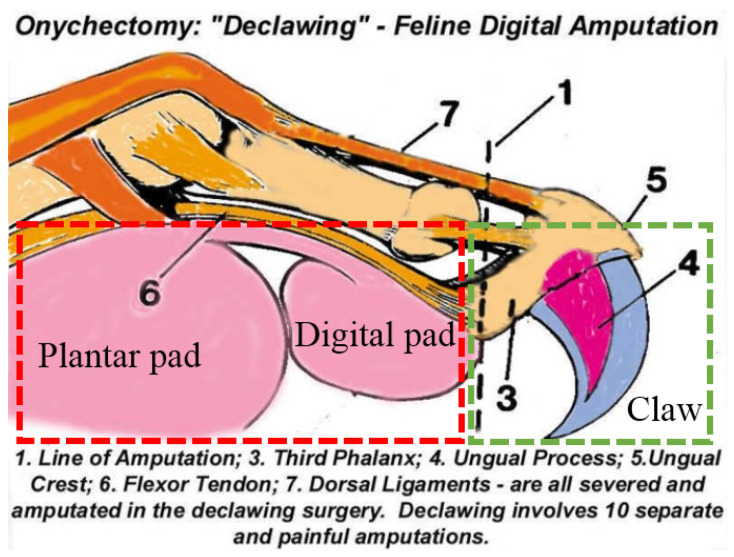
The skeletal structure of a cat claw [23].

As shown in Figure 2, the digital flexor tendon is at the bottom of the foot, and the third knuckles have claws. When the claws shrink, the third knuckles are towed by the elastic ligament and fold into the middle phalanx. If the toes in the cat leg flexor are shrunk, the claws are extended by the tensioning tendon driving the third knuckles. In this way, autonomously stretched claws can be achieved. The abilities of the “pads” to buffer and the claws to stretch autonomously and grip are also needed for the foot of a humanoid robot.

In bionics, the structure, form, function, energy conversion, information control, and other excellent features of biological systems are studied and applied to engineering and technology systems to improve existing equipment and provide innovative design, working principles, and system structures for engineering and technology. With the development of biology and bionics research, it has been discovered that a particular biological function is achieved through the synergy or coupling of many parts.

**Figure 3:**
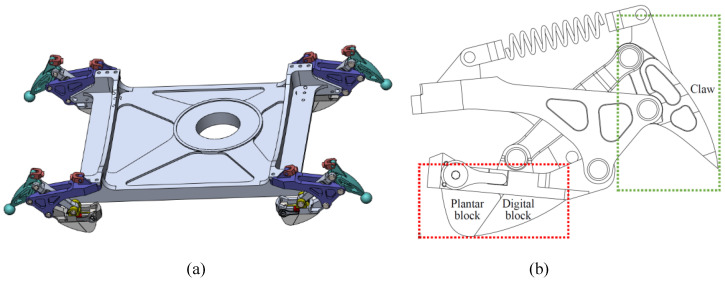
Humanoid robot foot and the bionic mechanism: (**a**). appearance of the foot with the bionic mechanism; (**b**) details of the bionic mechanism.

**Figure 4:**
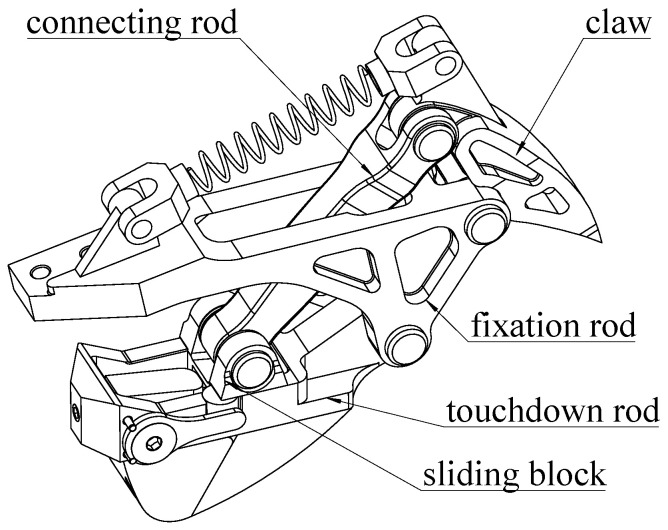
The bionic mechanism on the foot.

To increase the grip and buffering abilities, we designed the bionic mechanism on the humanoid robot foot as shown in Figure 3 by referencing the stretchable claws and plantar “pads” of the feline foot. Bionic mechanisms are fitted on foot corners. Increasing the distance to the buffer is achieved by a structure that has a four-bar linkage and a spring. Soft material is added to the part that contacts the ground to increase the ability to absorb the impact. The four rods are the touchdown rod, the claw, the fixation rod, and the connecting rod as shown in Figure 4.

**Figure 5:**
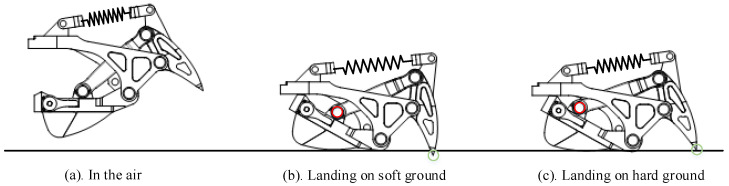
The different states of the bionic mechanism for different situations.

The touchdown rod is responsible for contacting the ground. The claw that penetrates the ground, as shown in Figure 5, plays a role in increasing grip. The fixation rod connects the foot with the bionic mechanism. The connecting rod links the claw with the touchdown rod. In this way, the claw in the bionic mechanism tends to penetrate the ground every time. When the humanoid robot with this type of foot walks on hard marble or tarmac for a long time, the claw is damaged and loses its function. In the interest of protecting the claw, the ability to shrink the claw when the robot is walking on hard ground is necessary. A slide is added on the touchdown rod to provide conditions for the end of the connecting rod to slide, and a limited block is used to restrict connecting rod and claw movements relative to the touchdown rod. Thereby, the claw posture is adjusted to protect itself. When the support foot moves away from the ground, the rubber blocks at the end of the slide can provide a restoring force for the slider to return to the initial position and be bounded by a limited block.

## 3. Optimization of Four-Bar Linkage

The four-bar linkage, the main bionic mechanism, is used to control the angle between the ground and claw and to absorb impact. The buffering and gripping performance of the four-bar linkage directly affects the entire mechanism function. Therefore, it is important to optimize the four-bar linkage in the mechanism design. Through the optimization of the parameters of each link in the bionic mechanism, it is possible to minimize the error between the theoretical trajectory and actual trajectory while ensuring that the structure is also compact and undergoes a lower force. Moreover, the angle of the claw penetrating the ground is a similarly important design parameter. The claw should be inserted into the ground at a vertical angle so that the bionic mechanism is only supported by vertical upward forces to maintain the stability of the robot. To ensure that the claw inserts into the ground vertically, we find a set of design parameters that can minimize the trajectory error. According to the constraints for the foot bionic mechanism, the optimal length of the link is obtained by theoretical calculation.

There are two important steps in the optimization. One is building a bionic mechanism, and the other is optimizing the lengths of the links. The optimization, design variables, and objective function are determined by comprehensively analyzing the bionic mechanism. First, we also set up constraints for the design of the bionic mechanism, such as the length of the rod and angle drive. Second, the constraints are transformed into the mathematical model and functions. Finally, an optimal design model of the bionic mechanism is created.

**Figure 6:**
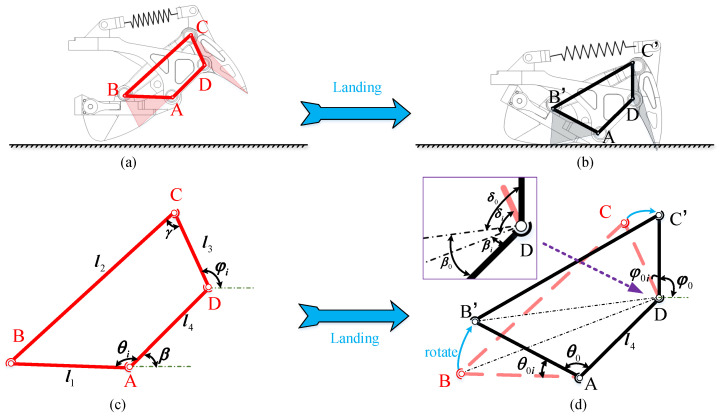
The four-bar linkage of the bionic mechanism. A, B, C, and D are the vertices of the four-bar linkage. BC is the connecting rod; AD is the fixation rod; CD is the claw; and B is the position of the sliding block. (**a**,**b**) describe the relationship between the four-bar linkage and the bionic mechanism during the landing motion. (**c**,**d**) show a state transition from “in the air” (ABCD) to “landing on soft ground” ($AB^{\prime }C^{\prime }D$), as defined in Figure 4. After the state switch is complete, the angle between the touchdown rod and the fixation rod changes from $\theta_{i}$ to $\theta_{0}$. Meanwhile, the angle between the claw and the fixation rod changes from $\varphi_{i}$ to $\varphi_{0}$. The black lines in the two subfigures indicate the same state of the bionic mechanism. $l_{1}$, $l_{2}$, $l_{3}$, and $l_{4}$ are the lengths of AB, BC, CD, and AD, respectively. Obviously, because the sliding block remains still, we have $AB^{\prime }=AB$, $B^{\prime }C^{\prime }=BC$, and $C^{\prime }D=CD$. The purple rectangle is an enlarged view around vertex D.

According to the bionic mechanism in Section 2, a typical four-bar linkage movement and the definitions of some parameters are shown in Figure 6. The touchdown rod plays the role of input. The given function is $\varphi_{0i}=f\left(\theta_{0i}\right)$, where $\varphi_{0i}=\varphi_{i}-\varphi_{0}$ is the rotation angle of AB ($∠BAB^{\prime }$) and $\theta_{0i}=\theta_{i}-\theta_{0}$ is the rotation angle of BD ($∠CDC^{\prime }$). The bionic mechanism states depend on seven variables, which are the length of the four-bar linkage $l_{1}$, $l_{2}$, $l_{3}$, $l_{4}$, the location of vertex A${\left(a_{x},a_{y}\right)}^{T}$, the angle β between the horizontal line and fixation rod AD, and the input angle $\theta_{0i}$. To simplify the problem, the following assumptions have been considered:

- β is constant;
- The position of A is ${\left(0,0\right)}^{T}$;
- $l_{4}=1$ (reference length of the fixation rod).

In this way, the number of variables decreases to four without affecting the function of the touchdown rod and claw. The function relationship between input $\theta_{0i}$ and output $\varphi_{0i}$ is obtained as follows:

(1) $$\varphi_{0i}=f\left(l_{1},l_{2},l_{3},\theta_{0},\theta_{0i}\right).$$

Vertical insertion of the claw into the ground to ensure that the lifting foot remains unhindered is the goal of bionic mechanism optimization. For optimization, we discretize the desired trajectory of $l_{3}$ to some points. Meanwhile, the rotation angle of the claw must be close to the given angle in all positions $\theta_{0i},i=1,2,\ldots ,s$. *s* is the number of position points of $l_{3}$, which is set by the user. Therefore, the optimization cost function of the four-bar linkage is expressed as follows:

(2) $$f\left(x\right)=\sum_{i=1}^{s}\omega_{i}{\left[f\left(l_{1},l_{2},l_{3},\theta_{0},\theta_{0i}\right)-g\left(\varphi_{0i}\right)\right]}^{2}$$

where $g(\varphi_{0i})$ is the target set by the user that the claw inserts vertically into the ground when the foot lands on the ground. As the angle of the touchdown rod is $\theta_{0i}$, the claw angle $\varphi_{0i}$ can be obtained as follows:

(3) $$\varphi_{0i}=\beta_{0}+\delta_{0}-\beta_{i}-\delta_{i}$$

with the residua,

$$\beta_{0}=\arctan\frac{l_{1}\sin\theta_{0}}{1-l_{1}\cos\theta_{0}}$$

$$\delta_{0}=\arccos\frac{l_{1}^{2}-l_{2}^{2}+l_{3}^{2}+1-2l_{1}\cos\theta_{0}}{2l_{3}\sqrt{l_{1}^{2}+1-2l_{1}\cos\theta_{0}}}$$

$$\beta_{i}=\arctan\frac{l_{1}\sin\left(\theta_{0}+\theta_{0i}\right)}{1-l_{1}\cos\left(\theta_{0}+\theta_{0i}\right)}$$

$$\delta_{i}=\arccos\frac{l_{1}^{2}-l_{2}^{2}+l_{3}^{2}+1-2l_{1}\cos\left(\theta_{0}+\theta_{0i}\right)}{2l_{3}\sqrt{l_{1}^{2}+1-2l_{1}\cos\left(\theta_{0}+\theta_{0i}\right)}}$$

where $\beta_{0}$ is $∠ADB^{\prime }$, $\delta_{0}$ is $∠B^{\prime }DC^{\prime }$, $\beta_{i}$ is ∠ADB, and $\delta_{i}$ is ∠BDC. Substituting Equation (3) into Equation (2) and defining $x={\left[x_{1},x_{2},x_{3},x_{4}\right]}^{⊤}$, which is representative of $l_{1}$, $l_{2}$, $l_{3}$, and $\theta_{0}$, the optimization objective function of the mechanism is obtained. The constraint conditions of the optimal design of the bionic mechanism are determined by the actual design situation. The bionic mechanism should be regarded as a crank rocker. Therefore, the following must be met:

(4) $$\left\{\begin{array}{c} 1-l_{1}-l_{2}+l_{3}\geq 0\\ 1-l_{1}+l_{2}-l_{3}\geq 0\\ -1-l_{1}+l_{2}+l_{3}\geq 0 \end{array}\right.$$

The bionic mechanism must be flexible and reliable; thus, the transmission angle γ (∠BCD) should meet the following conditions:

$${\left[\gamma \right]}_{\min}\leq \gamma \leq {\left[\gamma \right]}_{\max}$$

and

$$\cos{\left[\gamma \right]}_{\min}\leq \cos\gamma \leq \cos{\left[\gamma \right]}_{\max}$$

where

$$\cos\gamma =\frac{l_{2}^{2}+l_{3}^{2}-l_{1}^{2}-1+2l_{1}\cos\left(\theta_{0}+\theta_{0i}\right)}{2l_{2}l_{3}}$$

From the above formula, we know that the transmission angle γ changes with $\cos\left(\theta_{0}+\theta_{0i}\right)$. When $\cos\left(\theta_{0}+\theta_{0i}\right)$ is the maximum, γ is the minimum. When $\cos\left(\theta_{0}+\theta_{0i}\right)$ is the minimum, $\gamma_{i}$ is the maximum. Because of the crank rocker mechanism, ${\left[\cos\left(\theta_{0}+\theta_{0i}\right)\right]}_{\max}=1$ and ${\left[\cos\left(\theta_{0}+\theta_{0i}\right)\right]}_{\min}=-1$. To satisfy the above requirements, the constraint equations should satisfy the following conditions:

(5) $$\left\{\begin{array}{c} \cos{\left[\gamma \right]}_{\min}-\frac{l_{2}^{2}+l_{3}^{2}-{\left(1-l_{1}\right)}^{2}}{2l_{2}l_{3}}\geq 0\\ \frac{l_{2}^{2}+l_{3}^{2}-{\left(l_{1}+1\right)}^{2}}{2l_{2}l_{3}}-\cos{\left[\gamma \right]}_{\max}\geq 0 \end{array}\right.$$

The optimal design of the crank rocker mechanism uses a given function $g\left(\varphi_{0i}\right)=k_{a}\sin\left(k_{b}\theta_{0i}\right)$, where $k_{a}$ and $k_{b}$ are the positive values. We set $0<k_{b}\theta_{0i}<90^{\circ }$ to guarantee that $\varphi_{0i}$ increases monotonically as $\theta_{0i}$ increases. The derivative of the function sin is approximately equal to zero at approximately $90^{\circ }$. This ensures that the claw remains in a vertical position when penetrating the ground. The initial angles of the touchdown rod and claw are $\theta_{0}$ and $\varphi_{0}$, respectively. In this case, the rotation ranges of the touchdown rod and claw are $25^{\circ }$ and $35^{\circ }$, respectively. When the touchdown rod rotates from $\theta_{0}$ to $\theta_{0}+25^{\circ }$, the claw rotates from $\varphi_{0}$ to $\varphi_{0}+35^{\circ }$. Therefore, $k_{a}=35^{\circ }$ and $k_{b}=1/25^{\circ }$. Then, the cost function can be given as follows:

(6) $$f\left(x\right)=\sum_{i=1}^{s}{\left[\begin{array}{c} f\left(l_{1},l_{2},l_{3},\theta_{0},\theta_{0i}\right)\\ -35^{\circ }\sin\left(\frac{1}{25^{\circ }}\theta_{0i}\right) \end{array}\right]}^{2}$$

The optimization model of the four-bar linkage in the bionic mechanism is solved by the penalty function and Powell’s method. The penalty parameter is = $\gamma_{0}=0.001$ and the decreasing function parameter is e=0.01. Defining the initial point as $x^{\left(0\right)}={\left[l_{1},l_{2},l_{3},\theta_{0}\right]}^{T}={\left[1,2,0.7,100^{\circ }\right]}^{T}$, the actual length of the rod is entered into the cost function, and the length of the rod is shown in Table 1.

## 4. Design of the Limited Structure

The retracted claw can protect the foot. For a humanoid robot, a foot with a claw provides more grip when the robot walks on soft ground since the claw can penetrate the ground easily. However, when walking on hard ground, such as marble or asphalt, the claw on the foot is easily damaged if contact with the ground repeatedly occurs.

To solve this problem, shrinking the claw is a good means to protect the foot when the claw contacts hard ground. Meanwhile, the claw can penetrate soft ground. A slide mechanism with a limited block is designed to protect the claw.

As shown in Figure 4, the slider can move forward or backward on the slide. However, the movement is limited by a block, which is a small cylinder. For this reason, when the robot foot is in contact with the ground, the slider, which is limited by the cylinder, has difficulty moving backward and remaining stationary relative to the slide. At this time, the bionic mechanism is simply a four-bar linkage. The block under the slide contacts the ground, and the claw moves downward until it touches the ground. Elastic deformation of the spring can limit the movement of the limited block within a certain range, thereby limiting the movement of the slider. For a walking humanoid robot, the pressure on the claw becomes greatest during a single-support period since the entire weight of the robot is concentrated on one foot. On the one hand, the claw penetrates the ground when a humanoid robot is walking on soft ground. On the other hand, when the claw contacts hard ground, the pressure of the claw is greater than the pressure when walking on soft ground. The limited block and the elastic deformation of the spring become larger because of the increasing force of the slider. When the deformation reaches a certain value, the slider breaks through the limited block and moves to the end of the slide.

**Figure 7:**
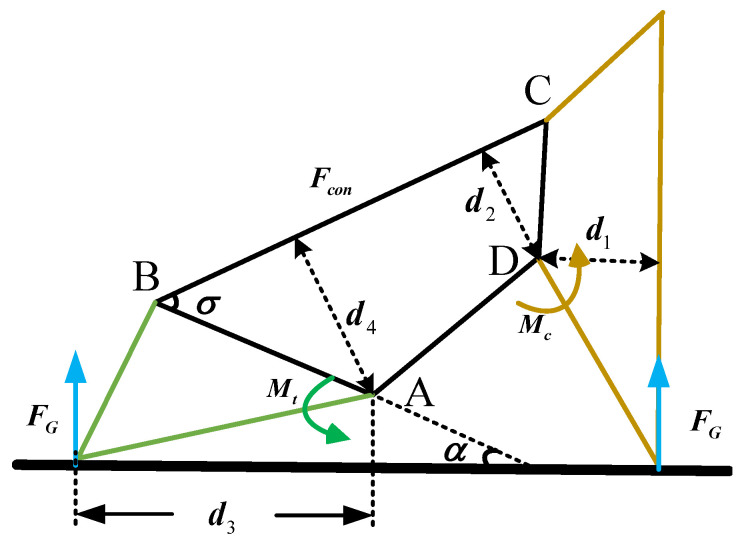
The state of the bionic mechanism when the foot lands on the ground. $F_{G}$ is the support from the ground. $F_{con}$ indicates the internal force of the connecting rod. $M_{t}$ denotes the moment of the touchdown rod with respect to point A defined in Figure 6; $M_{c}$ denotes the moment of the claw with respect to point D. α is the angle between the touchdown rod and the horizontal line. σ is ∠ABC. $d_{1}$ and $d_{3}$ are the distances from D and A to the extension of force $F_{G}$, respectively. $d_{2}$ and $d_{4}$ are the distances from D and A to the connecting rod CD, respectively.

According to the static analysis of the four-bar linkage, the pressure in the cylinder top is calculated, which is convenient for finite element analysis in the following subsection. The total weight of the robot is 50 kg. Both the robotic feet touch the ground, and the bionic mechanism state is shown in Figure 7.

At this time, the eight claws are stressed simultaneously. The torque couple of every rod is zero. The forces of the slides and claws are analyzed. The torque of the slide and claw relative to the corresponding fixation rods is zero; therefore, we have the following:

(7) $$\left\{\begin{array}{c} M_{c}=F_{G}\times d_{1}+F_{con}\times d_{2}\\ M_{t}=F_{G}\times d_{3}+F_{con}\times d_{4} \end{array}\right.$$

The stress of the limit block on the slide can be given as follows:

$$F_{all}=F_{con}\times \cos\left(\sigma \right)+F_{G}\times \sin\left(\alpha \right)\approx 100N$$

### Finite Element Analysis

**Figure 8:**
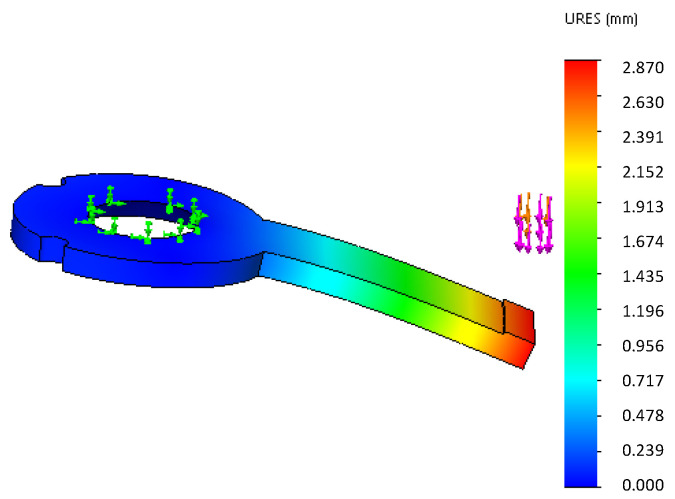
The finite element analysis result of the springs.

One end of the steel spring is connected with the slide by screws, while the other end is not fixed to the limited block. When the foot contacts the ground, the claw tip is subjected to impact by the ground, and the impact is transferred through the four-bar linkage mechanism. The limited block undergoes elastic deformation. As shown in Figure 8, the green arrow is the fixed object.

The purple arrow indicates the force exerted on the spring. The pressure of the limited block is 100 N, which is calculated in Section 3. Then, the appropriate materials are selected and shown in Table 2.

From the finite element analysis results, the deformation of the model’s “4×0.8” and “5×0.8” springs appeared to be more appropriate when the pressure reached 100 N. The deformation of both springs can allow the limited block to be pushed out by the slider, and the spring remains in the range of the bearing.

Moreover, the claw will be affected by the impact when it lands on the ground. The impact force must be greater than the static analysis results, and the impact force is difficult to obtain by calculation. However, it is easy to obtain more accurate results through dynamic simulation. Due to the great impact force, finite element analysis for the claw is necessary; however, the finite element analysis process is not repeated here for simplification.

## 5. Simulation

**Figure 9:**
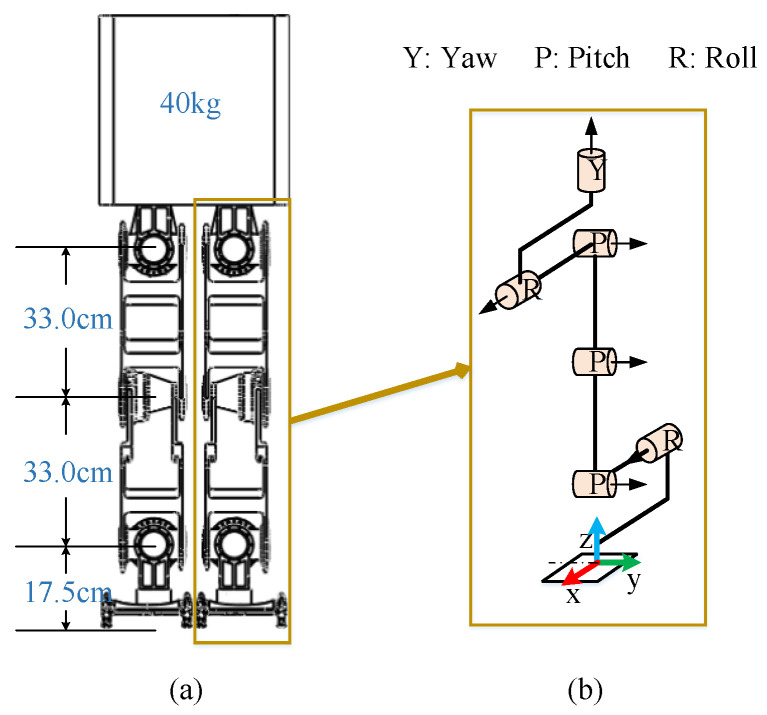
The humanoid robot platform: (**a**) the length of the leg and the weight of the trunk; (**b**) configuration of the DOFs of a leg and the direction of the frame.

Dynamic simulation can help us to find any structural problems during the design phase. It has great significance in the design of a humanoid robot. In the simulation, it is necessary to ensure that the simulation environment is as similar as possible to the actual situation. To improve the reliability and authenticity of the model, the robot model in this paper is built to match the length, weight, and DoF data of the actual humanoid robot as shown in Figure 9.

**Figure 10:**
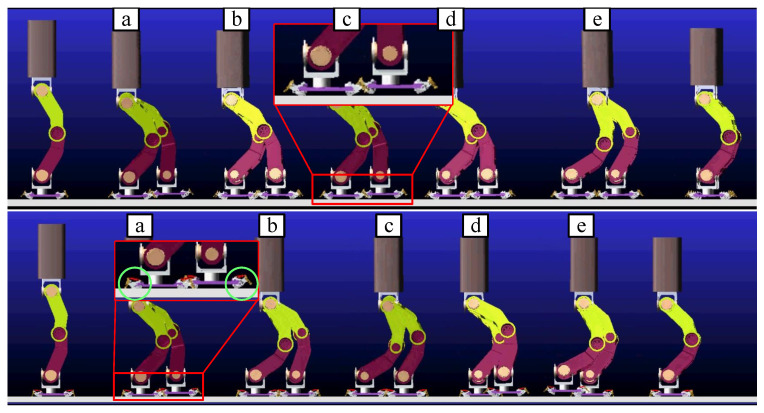
The dynamics simulation of a walking humanoid robot. The dynamics simulation of a walking humanoid robot. The red boxes indicate the partial enlargement of the foot. The green circles show the bionic mechanism in different states on different feet.

As shown in Figure 10, the four-bar linkage for bionic mechanism movement is good in ADAMS simulation.

**Figure 11:**
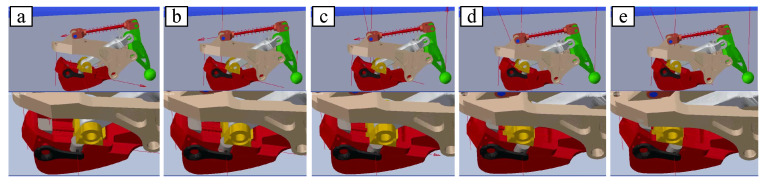
The snapshots of the bionic mechanism simulation. The fixation rod is constrained to move only vertically up and down. As the downward pressure increases, the slider pushes away from the cylinder and moves backwards on the rail.

On the one hand, when the humanoid robot foot contacts the ground, the bionic mechanism can play a buffer role to avoid the flat foot directly contacting the ground. Direct foot contact with the ground will cause a large impact. In particular, the robot will topple over due to the large impact generated by the toes landing on the ground prematurely. In other words, if the swinging foot lands on the ground faster than the designed time of the planned dynamic pattern, then the moment of tipping backward occurs, and the robot will tip backward [24]. On the other hand, the long-term contact between the ground and the foot can also achieve the purpose of increasing stability because the posture of the foot can be estimated by force sensors as the bionic mechanism landing before the foot touches the ground firmly. As the upper subfigure of Figure 10 shows, the bionic mechanism is fixed on the foot firmly to simulate walking by foot, so the foot lands by treating the four points as a surface (flat foot). During this process, the bionic mechanism does not work. The claw is suspended in air, as shown in the red block of the upper subfigure, rather than moving downward to insert into the ground. Meanwhile, as shown in the lower subfigure of Figure 10, the bionic mechanisms inside two green circles are in two different states, i.e., “landing on soft ground” and “in the air”, as defined in Figure 4, corresponding to the support leg and the swinging leg, respectively. When the swinging leg lands on the ground, the blocks under the touchdown rod are the first to land, followed by tensioning of the spring and the claw contacting the ground. As shown in Figure 11, the force on the claw pushes the slider on the rail as the vertical downward force gradually increases.

### 5.1. Buffering Capacity

**Figure 12:**
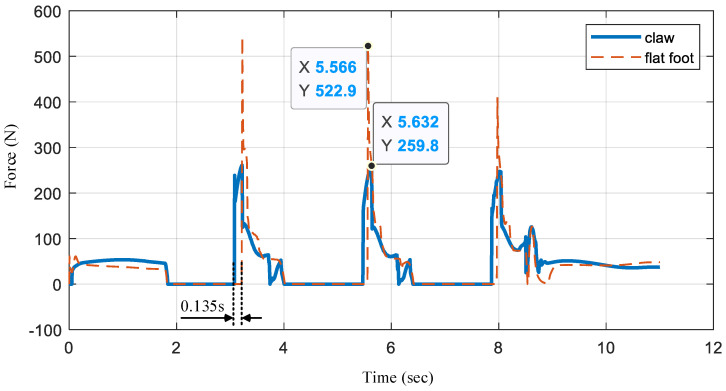
The comparison results of impact with the bionic mechanism.

As shown in Figure 12, we record the force of the bionic mechanism in the simulation (in Figure 10). When the foot is in the air, the force is 0 N. We analyze the impact force of the three landings. When the foot is landing without the bionic mechanism working (flat foot), the force (red dashed line) increases from 0 N to 522.9 N. Meanwhile, when the bionic mechanism works, it continues to move downward after the digital block contacts the ground, as described in Figure 4. Therefore, the time of contact with the ground is 0.135 s longer. This longer contact time provides more adjustment time for admittance control for landing. In addition, the impact of the foot with the bionic mechanism is nearly half that with a flat foot.

### 5.2. Stability

**Figure 13:**
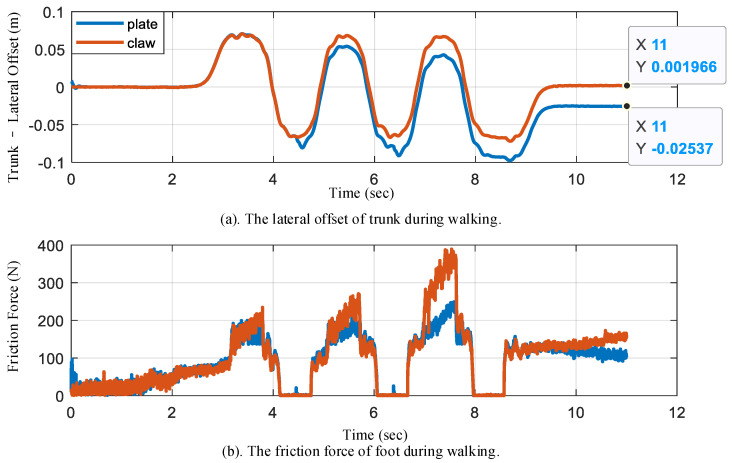
A humanoid robot walks with two different feet (flat foot and foot with bionic mechanism) in a simulation. We compare the lateral offset of the trunk and the friction force of the foot: (**a**) the lateral offset of trunk; (**b**) the friction force of the foot.

In the simulation, we can obtain the robot walking trajectory with two different feet (flat foot or foot with four bionic mechanisms). Figure 13 shows the lateral offset and friction force of humanoid robot walking. Since the mass of the robot is mainly concentrated in the weight above the hip, the center of the weight mass is approximately at the centroid of the humanoid robot. During walking in a straight line, the left foot and right foot alternately touch the ground, and the center of mass (CoM) is controlled to move from one side to the other to remain stable. As shown in the upper subfigure of Figure 13a, the lateral position of the trunk (an approximation of the center of mass.) oscillates up and down around zero depending on the step frequency. For example, when the lateral position is positive, the left foot is the main supporting leg. As shown in the lower subfigure of Figure 13b, when the friction force is zero, the left foot swings in the air. The friction forces of the foot with the bionic mechanism are greater than those without the bionic mechanism, especially on the second and third foot landings. The greater friction can resist the torque caused by the swinging leg, resulting in a small lateral offset when walking straight. With the bionic mechanism, the lateral offset of the humanoid robot is close to 0.2 cm. However, without the bionic mechanism, the lateral offset is more than 2.5 cm. The lateral offset of the humanoid robot is significantly reduced, which allows the goal of increasing stability to be achieved, because the bionic mechanism provides more friction force, as shown in Figure 13b.

## 6. Conclusions

In this paper, we proposed a new humanoid foot that consists of four bionic mechanisms located on each corner. The bionic mechanism is inspired by a felid, with retractable claws and fleshy pads to improve the traction and cushioning capabilities. The bionic mechanism consists of a self-recovery (using a spring) four-bar linkage and a slider that protect the claw in the situation of contacting hard ground. The proposed foot can improve the buffering and stabilizing ability. Finally, the feasibility of the foot mechanism has been verified with a dynamic simulation.

## Figures and Tables

**Table 1:** The length of each rod.

Rod Name	Length (cm)
Fixation rod	2.12
Touchdown rod	2.25
Connecting rod	4.12
Claw	1.51

**Table 2:** Material properties.

Material	65 Mn spring steel
Elastic Modulus (Young’s modulus)	$1.9\times 10^{5}$ (MPa)
Tensile Strength	980 (MPa)
Yield Strength	785 (MPa)

## Data Availability

Not applicable.

## References

[1] Yu Z., Fu J., Ji Y., Zhao B., Ji A. (2022). Design of a Variable Stiffness Gecko-Inspired Foot and Adhesion Performance Test on Flexible Surface. Biomimetics.

[2] Hong S., Park G., Lee Y., Lee W., Choi B., Sim O., Oh J.H. (2018). Development of a tele-operated rescue robot for a disaster response. Int. J. Humanoid Robot..

[3] Sohn K. (2019). Optimization of vehicle mounting motions and its application to full-sized humanoid, DRC-Hubo. J. Intell. Robot. Syst..

[4] Shigemi S., Goswami A., Vadakkepat P. (2019). ASIMO and humanoid robot research at Honda. Humanoid Robotics: A Reference.

[5] Narang G., Kong W., Xu P., Narang A., Singh S., Hashimoto K., Zecca M., Takanishi A. Comparison of bipedal humanoid walking with human being using inertial measurement units and force-torque sensors. Proceedings of the 2013 IEEE/SICE International Symposium on System Integration.

[6] Yu L., Zhao J., Ma Z., Wang W., Yan S., Jin Y., Fang Y. (2022). Experimental Verification on Steering Flight of Honeybee by Electrical Stimulation. Cyborg Bionic Syst..

[7] Wang L., Meng L., Kang R., Liu B., Gu S., Zhang Z., Meng F., Ming A. (2022). Design and Dynamic Locomotion Control of Quadruped Robot with Perception-Less Terrain Adaptation. Cyborg Bionic Syst..

[8] Nagasaka K. Stabilization of dynamic walk on a humanoid using torso position compliance control. Proceedings of the 17th Annual Conference on Robotics Society of Japan.

[9] Takenaka T., Matsumoto T., Yoshiike T., Hasegawa T., Shirokura S., Kaneko H., Orita A. Real time motion generation and control for biped robot-4 th report: Integrated balance control. Proceedings of the 2009 IEEE/RSJ International Conference on Intelligent Robots and Systems.

[10] Huang Q., Dong C., Yu Z., Chen X., Li Q., Chen H., Liu H. (2022). Resistant Compliance Control for Biped Robot Inspired by Humanlike Behavior. IEEE/ASME Trans. Mechatron..

[11] Seiwald P., Wu S.C., Sygulla F., Berninger T.F., Staufenberg N.S., Sattler M.F., Neuburger N., Rixen D., Tombari F. LOLA v1. 1–An Upgrade in Hardware and Software Design for Dynamic Multi-Contact Locomotion. Proceedings of the 2020 IEEE-RAS 20th International Conference on Humanoid Robots (Humanoids).

[12] Davis S., Caldwell D.G. The design of an anthropomorphic dexterous humanoid foot. Proceedings of the 2010 IEEE/RSJ International Conference on Intelligent Robots and Systems.

[13] Huang Q., Zhang W., Li J., Han C. (2009). Humanoid Robot Foot Section Impact Absorption Mechanism.

[14] Buschmann T., Lohmeier S., Ulbrich H. (2009). Humanoid robot lola: Design and walking control. J.-Physiol.-Paris.

[15] Oh P., Sohn K., Jang G., Jun Y., Ahn D., Shin J., Cho B.K. (2018). Team DRC-Hubo@ UNLV in 2015 DARPA robotics challenge finals. The DARPA Robotics Challenge Finals: Humanoid Robots to The Rescue.

[16] Park I.W., Kim J.Y., Oh J.H. Online biped walking pattern generation for humanoid robot khr-3 (kaist humanoid robot-3: Hubo). Proceedings of the 2006 6th IEEE-RAS International Conference on Humanoid Robots.

[17] Li J., Huang Q., Zhang W., Yu Z., Li K. Flexible foot design for a humanoid robot. Proceedings of the 2008 IEEE International Conference on Automation and Logistics.

[18] She H., Zhang W., Huang H., Yu Z., Chen X., Huang Q. Anti-skid foot design for a humanoid robot. Proceedings of the 2014 IEEE International Conference on Robotics and Biomimetics (ROBIO 2014).

[19] Narioka K., Homma T., Hosoda K. Humanlike ankle-foot complex for a biped robot. Proceedings of the 2012 12th IEEE-RAS International Conference on Humanoid Robots (Humanoids 2012).

[20] Kim G.S., Yoon J. Development of intelligent foot with six-axis force/moment sensors for humanoid robot. Proceedings of the 2008 IEEE International Conference on Robotics and Biomimetics.

[21] Liu H., Huang Q., Zhang W., Chen X., Yu Z., Meng L., Bao L., Ming A., Huang Y., Hashimoto K. Cat-inspired mechanical design of self-adaptive toes for a legged robot. Proceedings of the 2016 IEEE/RSJ International Conference on Intelligent Robots and Systems (IROS).

[22] Fukuda T. (2020). Cyborg and Bionic Systems: Signposting the Future. Cyborg Bionic Syst..

[23] Feline Digital Amputation—“Onychectomy”. http://www.rchsks.org/archives/i-want-to/suggested-reading/the-facts-about-declawing.

[24] Huang Q., Nakamura Y. (2005). Sensory reflex control for humanoid walking. IEEE Trans. Robot..

